# The administration of Exendin-4 and CCK affects food intake differentially in female and male rats tested on an alternate day fasting paradigm

**DOI:** 10.1016/j.neulet.2023.137275

**Published:** 2023-04-26

**Authors:** Taityana J. Lopez, Marc A. Barcelos, Yada Treesukosol

**Affiliations:** Department of Psychology, California State University Long Beach, Long Beach CA 90840, USA

**Keywords:** High fat diet, GLP-1, Diet preference, Sex difference, Satiety, Hedonics

## Abstract

Alternate day fasting (ADF) which involves the repetition of a 2-day cycle of a day of free access to food followed by a day of limited or no access to food, is an effective dietary intervention for weight loss in both humans and rats. We have previously reported that when presented with a high energy (HE) and standard chow diet, rats maintained on an ADF schedule displayed decreased HE diet preference compared to controls. Both male and female ADF rats increased overall intake of chow. However, this increase was driven by both meal size and meal number for males and only number of meals for females. Administration of cholecystokinin (CCK) or the glucagon-like peptide 1 (GLP-1) receptor agonist Exendin-4 (Ex-4) reduces food intake. It appears that CCK decreases food intake primarily through satiety signals whereas GLP-1 signaling may reduce intake by satiety and reward cues. Here, female and male rats were administered (i.p.) saline, 3.0 μg/kg Ex-4 (3 h before test), 3.0 μg/kg CCK (15 min before test) or a combination of both. Next, all rats were presented 23-h access to both HE diet and chow following food-restriction (ADF) or free access to chow (CON). Compared to saline-control sessions, administration of the combination of Ex-4 and CCK, but not Ex-4 or CCK alone, resulted in a decrease in both HE and chow intake early in the session for male ADF rats but the combination primarily decreased chow diet intake early in the session for female ADF rats. Thus, it appears that under these energy homeostatic conditions, administration of Ex-4 or CCK alone does not affect intake in ADF rats, but the combination produces decreases in feeding that are more than the sum of their individual effects. These findings support a role for the combination of GLP-1 and CCK signaling in the changes in diet preference induced by an alternate day fasting paradigm differentially in female and male rats.

## 1. Introduction

In the diet obesity model, rodents presented a palatable, calorically dense high energy (HE) diet will overeat and gain weight. This model provides an experimental analogy for the overconsumption of calorie-dense foods that contribute to overweight and obesity observed in the human population. The presentation of HE has been associated with hyperphagia driven by larger meal size [22,24,51,71]. Further, the presentation of HE and other food options, a model more comparable to conditions in humans, may amplify the HE-diet induced hyperphagia [41,59].

Alternate day fasting (ADF) is a dietary intervention that involves the repetition of a period of no or limited access to food followed by a period of ad libitum access to food. In humans, ADF has been shown to improve several measures associated with cardiometabolic risk such as body weight, BMI, blood glucose, triglyceride concentrations, cholesterol [8,75] and biomarkers of inflammation associated with obesity [7]. An ADF regime has also been shown to lead to weight loss without unsafe or aversive outcomes [25,35,56], even when participants are presented a high fat diet regime [40]. In rodent models, in addition to weight loss, ADF has led to a number of beneficial health outcomes including glucose and insulin regulation [2,13,33,39], lifespan prolongation [6,69] and mitigation of cognitive deficits associated with poor physical health [65,76].

Previously we reported that when presented HE and chow diets, rats maintained on an ADF schedule displayed lowered HE diet preference, compared to control groups. Both male and female ADF rats increased overall chow intake. However, for male ADF rats, the decrease in HE preference was driven by both an increase in chow meal size and meal number, whereas for females the change in preference was driven only by an increase in number of chow meals. Furthermore, for male ADF rats, the shift in diet preference was also driven by a decrease in HE caloric intake. Given that meal size is controlled by both positive feedback (e.g. from the oral cavity) and negative feedback (e.g. from postoral inhibitory signals) [15–17,49,66], it appears that for males, fasting increases orosensory stimulation and/or decreases sensitivity to inhibitory cues towards chow. For females, fasting appears to dampen sensitivity to postingestive inhibitory cues, a form of negative feedback that attributes to food intake reduction [16,17] towards chow. Thus, alternate day fasting paradigms appear to differentially affect females and males.

Several peptides secreted in the gastrointestinal tract are involved in the regulation of food intake. Notable among these for the present experiment are cholecystokinin (CCK) and glucagon-like peptide 1 (GLP-1). The alterations of CCK and GLP-1 secretion in response to feeding are consistent with their roles in satiety [see [53]]. When products of digestion reach the lumen, CCK is secreted from the duodenum and drives satiation. The administration of CCK decreases food intake by reductions in meal size in a dose-dependent manner [28,46] and the antagonism of CCK receptors enhance food intake by increases in meal size [54]. In response to feeding, GLP-1 is secreted from intestinal L cells [9,36] and appears to reduce food intake through both satiety and reward-related feeding pathways. Neurons that produce GLP-1 innervate regions involved in energy homeostatic control such as the hypothalamus and nucleus of the solitary tract [30,42] which have been documented to express GLP-1 receptor [52]. Expression of GLP-1 receptors has also been observed in the ventral tegmental area and nucleus accumbens, key regions of the mesolimbic pathway and have been shown to be involved in hedonic drive of food intake [19]. Activation of GLP-1 receptors in the nucleus solitary tract has also been shown to affect food reward behavior by activity in the mesolimbic system [58]. Administration of GLP-1 reduces meal size yet GLP-1 receptor antagonists fail to increase meal size [62] thus it is unlikely that GLP-1 plays a physiological role in meal termination. Taken together, it appears that CCK and GLP-1 regulate food intake by different mechanisms. Here, female and male rats were presented chow and HE diet following food-restriction or ad libitum access to chow to investigate the role of CCK and GLP-1 signaling on shifts in diet preference induced by alternate day fasting.

## 2. Methods

### 2.1. Subjects

A total of 24 female and 24 male Sprague Dawley rats were used in the behavioral test. The rats were raised at California State University, Long Beach. Rats were obtained from the litters of breeders purchased from Charles River (Hollister, CA). Litters were culled to 10 pups on postnatal day 3 and kept with the dam until weaning on postnatal day 21. After weaning, rats were group housed with same-sex littermates. Housing was in a temperature-controlled room maintained on a 12 h-12 h light–dark cycle. All procedures were approved by the Institutional Animal Care and Use Committee at California State University, Long Beach.

### 2.2. Behavioral procedure

Rats were single housed in DietMax System food intake monitoring cages (AccuScan Instruments, Inc., Columbus OH). The clear, polycarbonate cages were 42 × 42 × 30 cm (length × width × height) with a solid base. Two openings (6.5 × 6.5 cm) in the cage wall 11.2 cm apart, each provided access to food jars, which were placed on scales. Rats received ad libitum access to powdered chow (3.1 kcal/g, calories from protein 24%, calories from fat 18%, calories from carbohydrate 58%; PicoLab Rodent Diet 20, Lab Diet) unless otherwise noted.

After at least 5 days of habituation, rats were assigned to one of two groups (ADF or CON) such that there were no significant group differences in body weight and daily intake. Rats in the ADF condition were food-restricted for 24 h followed by 23-h access to standard chow and HE diet (4.73 kcal/g; calories from protein 20%, calories from fat 45%, calories from carbohydrate 35%; D12451, Research Diets). Rats in the CON (control) condition had ad libitum access to the standard chow every day with the addition of ad-libitum access to the HE diet every other day. Days on which rats were presented both the chow and HE diets are referred to as the testing days. On testing days, all rats were injected i.p. with saline, 3 μg/kg Ex-4 3 h before the test session and/or 3 μg/kg CCK 15 min before access to both the chow and HE diets (Table 1).

Rats were housed in the food intake monitoring cages undisturbed for 23-h test sessions. During this time, food intake was constantly recorded by weight fluctuations of the scales holding the food containers using the computer program Fusion 6.0 DietMax. For approximately 1 h each day, no data from the scales was recorded. During this time, rats were weighed and vaginal smears from female rats were recorded to account for estrous cycles. Water bottles were weighed to measure daily water intake. Food was refilled or removed daily according to the experimental condition.

## 3. Data analyses

Across the baseline test days, preference for the HE diet was expressed as the percentage of HE calories consumed of the total caloric (HE and chow) intake at the end of the 23-h session. To compare high energy diet preference values between ADF and CON groups across the three saline-injection baseline days, separate two-way ANOVAs (group and session) were conducted for females and males. Cumulative caloric intake of each diet was calculated across each 15-min time bin across the 23-h test sessions. To determine the time points at which caloric intake of a given diet between ADF and CON groups were comparable, a two-sample *t*-test was conducted followed by calculation of area under the curve as a measure of rate of intake. To determine the point at which chow and HE caloric intake differed for a given group, paired t-tests were conducted. To investigate the effects of peptide administration on HE and chow intake, cumulative intake across the first 90 and 330 min was compared with values from each previous saline-injected test day to take into account any potential changes in intake as a function of diet exposure. On some test days when food spilled from the scale containers, data from both foods were excluded from analysis for that animal for that day.

## 4. Results

### 4.1. Baseline measures

**Fig. 1.**
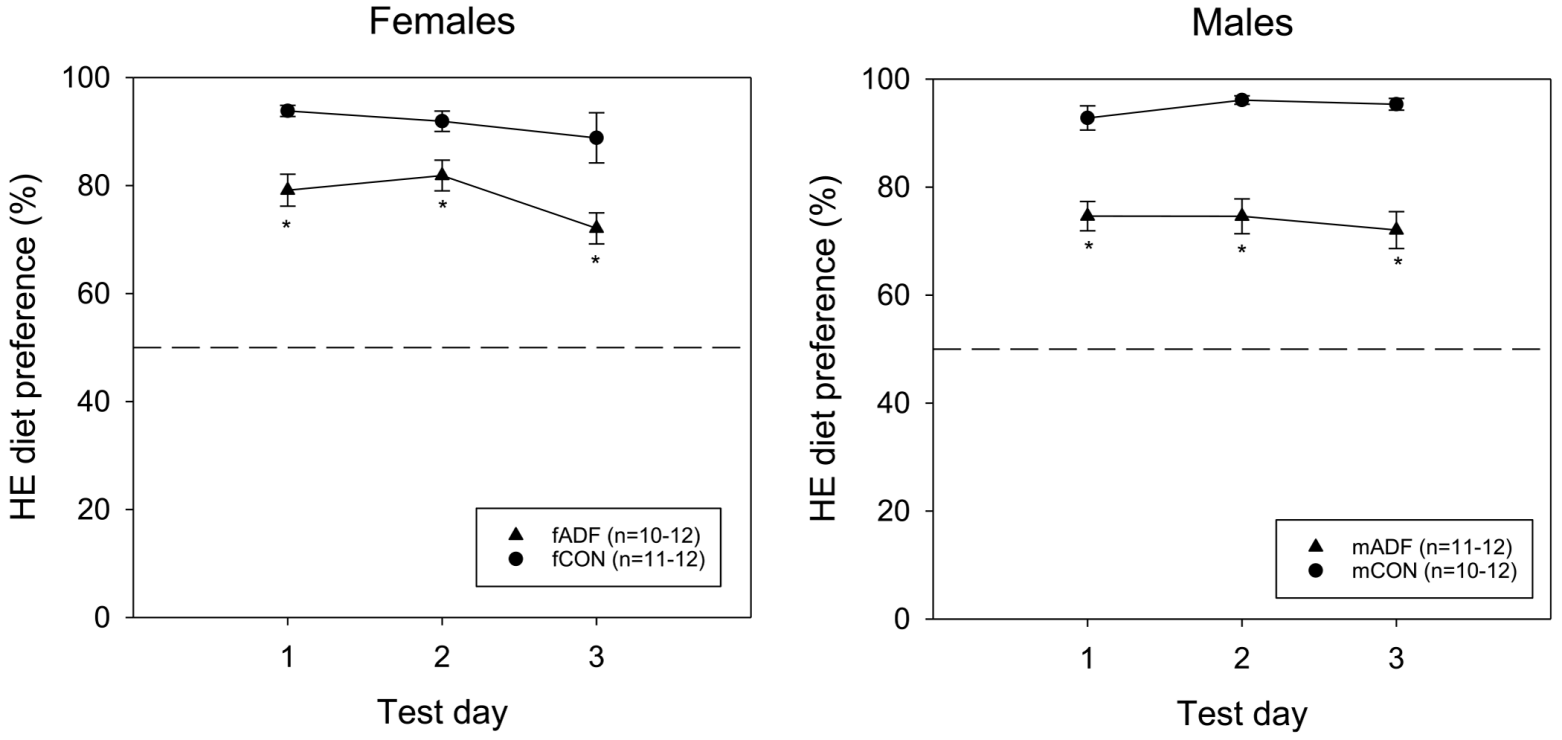
HE diet preference ± SE calculated as HE diet caloric intake by overall caloric intake across the first 3 baseline 23-h test sessions for female and male ADF and CON groups. * denotes significant group differences p < 0.05.

Consistent with our previous reports, female ADF rats showed significantly lower HE diet preference than their same-sex control group across the baseline saline-sessions (Fig. 1). A two-way ANOVA comparing HE diet preference at the end of each 23-h session across these sessions revealed a main effect of group (F(1,18) = 12.614, p = 0.002), a main effect of session (F(2,36) = 4.098, p = 0.025) and no interaction effect (F(2,36) = 0.755, p = 0.477). Post-hoc Bonferroni corrections revealed group differences at each time point and preference on test day 3 was lower than that on test day 2. Similarly, male ADF rats showed significantly lower HE diet preference than male CON. A two-way ANOVA comparing the preference values across baseline sessions revealed a main effect of group (F(1,19) = 40.244, p < 0.001), no main effect of session (F(2,38) = 1.771, p = 0.184) and no interaction effect (F (2,38) = 3.149, p = 0.054). Post-hoc Bonferroni corrections revealed group differences on each test day.

**Fig. 2.**
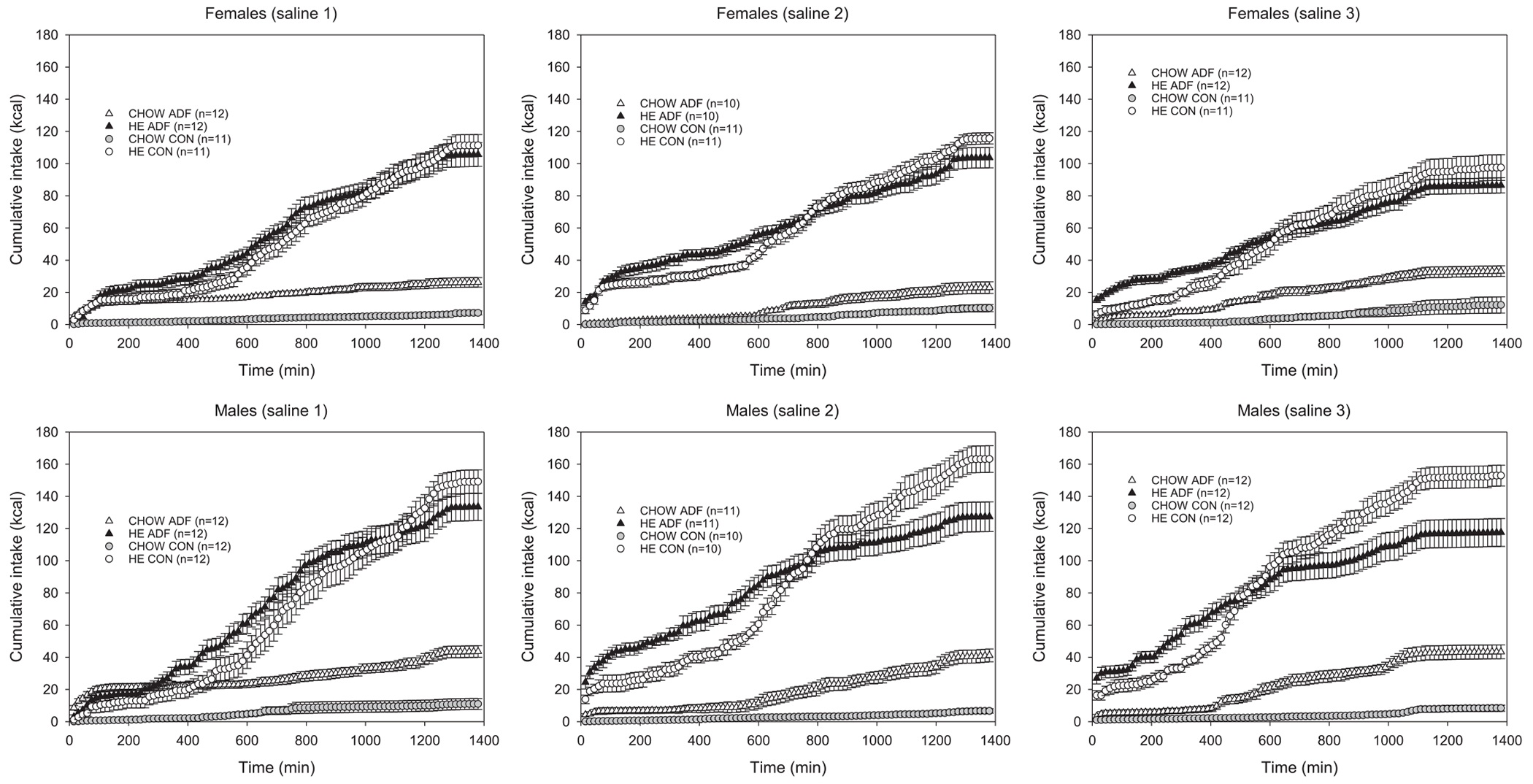
Cumulative caloric intake ± SE for chow and HE across each 15-min time bin across the first 3 baseline 23-h test sessions for female and male ADF and CON groups. All rats were presented both the chow and HE diets across these test sessions.

Cumulative intake values across the 23-h sessions reveal that upon the first presentation of HE diet with familiar standard chow, both female and male CON rats primarily eat the novel HE diet throughout the session. In contrast, caloric intake from both diets is comparable for the first hours of the session for ADF rats (Fig. 2, saline 1). Specifically, paired t-tests comparing cumulative caloric intake of the two diets reveal intake from both diets is similar until the 135-min time point for ADF females (t(12) = −2.106, p = 0.059) and until the 345-min time point for ADF males (t(11) = −1.460, p = 0.172). In contrast, from the second exposure to HE diet onwards, all groups eat more HE diet than chow from the start of the session. The area under the curve (AUC) of cumulative intake was compared as a measure of rate of intake. During the second test session at the 615-min time point, a two-sample *t*-test revealed HE cumulative intake was comparable between the two female groups (t(19) = 1.715, p = 0.103), but ADF females displayed a higher rate of HE intake than their respective controls (t(19) = 2.671, p = 0.015) across this period. Similarly for males during the second test session, a two-sample *t*-test revealed HE cumulative intake was similar between male ADF and CON groups (t(19) = 1.311, p = 0.205) at the 660-min time point and comparisons of AUC revealed male ADF rats displayed a higher rate of intake than male CON (t(19) = 3.529, p = 0.002) (Fig. 2, saline 2). This pattern of intake is observed in subsequent sessions.

### 4.2. Effect of GLP-1 and CCK receptor agonist administration

**Fig. 3.**
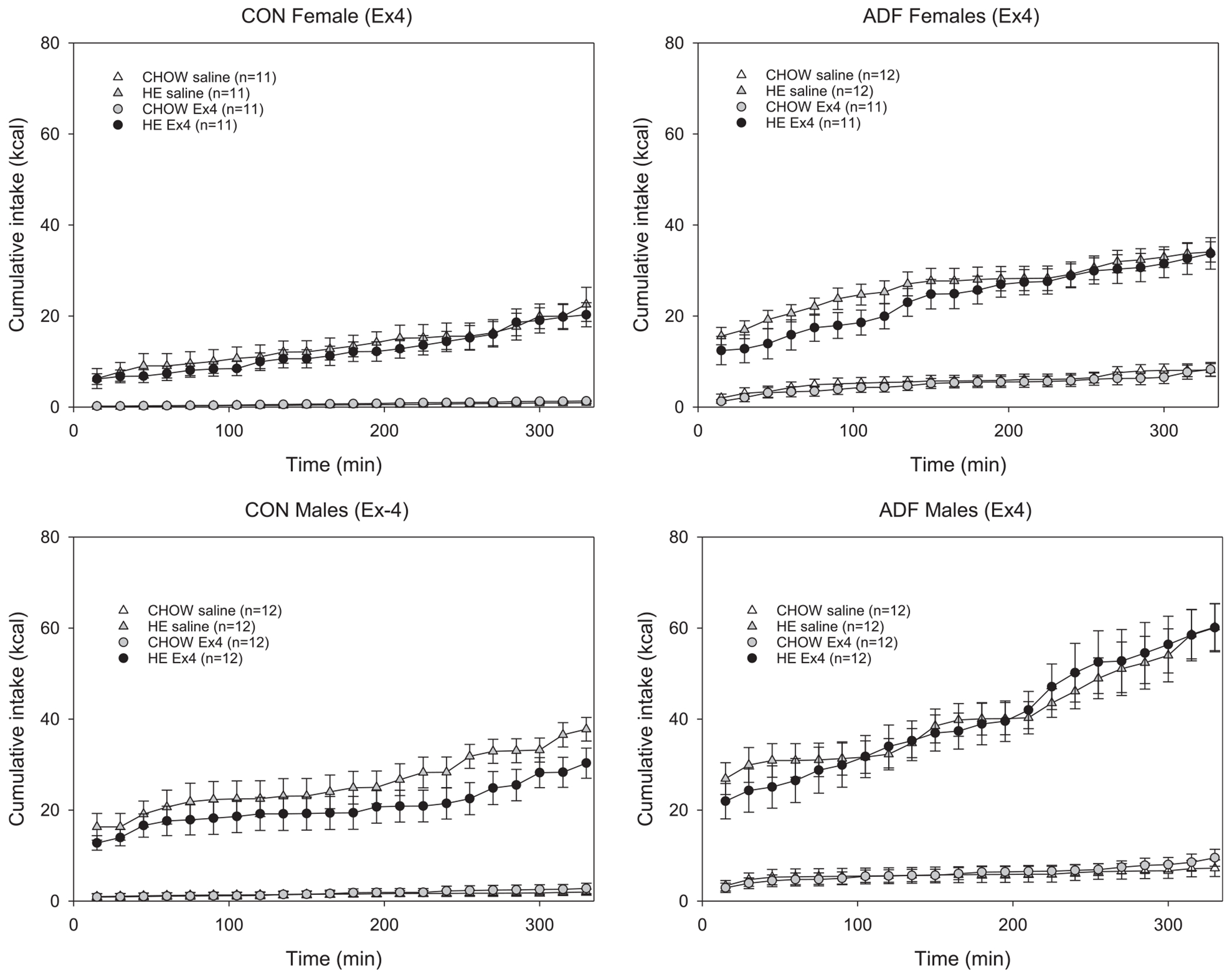
Cumulative caloric intake ± SE for chow and HE for female (top row) and male (bottom row) control (left column) and ADF (right column) groups across the first several hours. All rats were presented both the chow and HE diets across these test sessions and comparisons between responses following 3.0 μg/kg Ex-4 and the previous saline-injected session are shown in each graph.

**Fig. 4.**
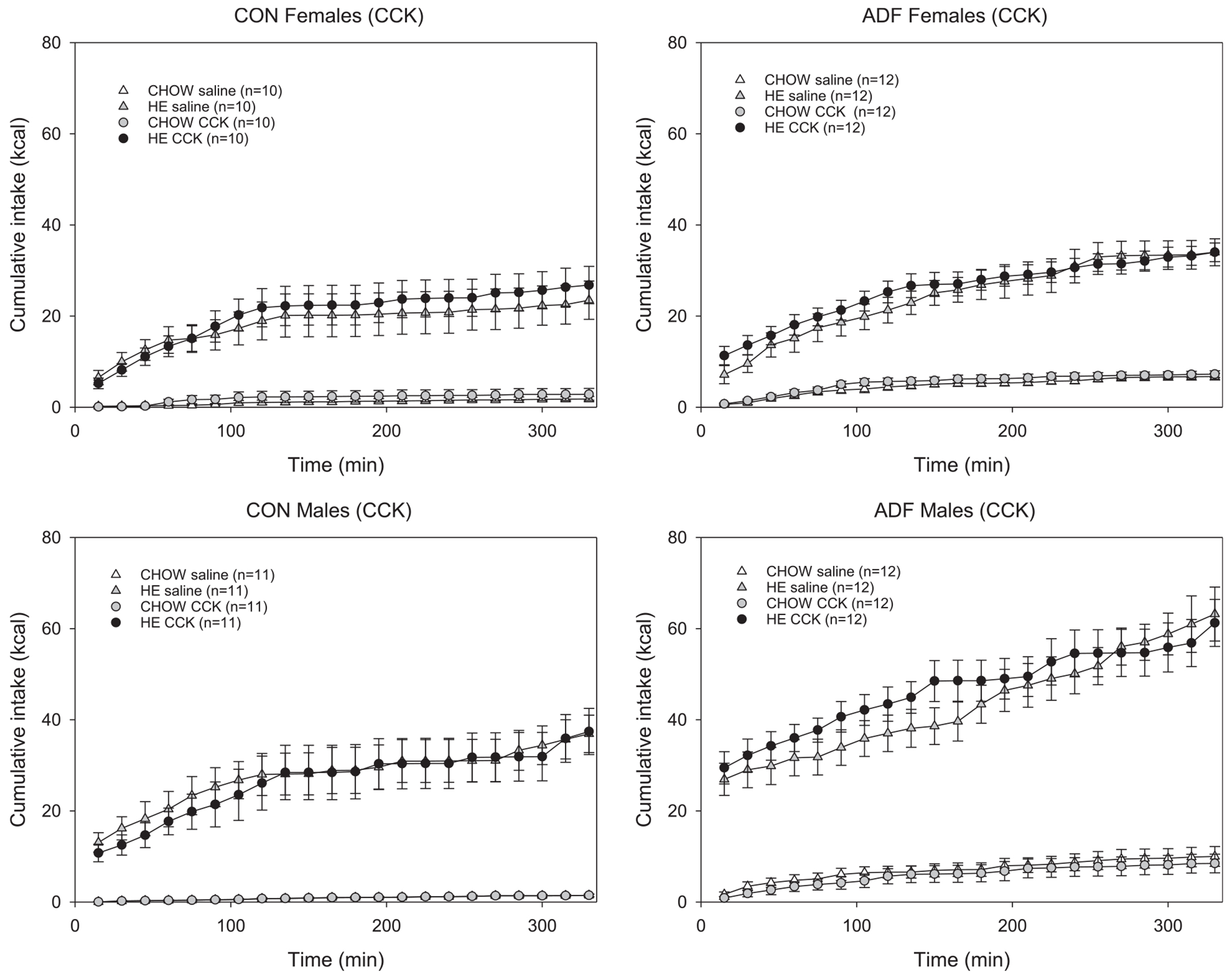
Cumulative caloric intake ± SE for chow and HE for female (top row) and male (bottom row) control (left column) and ADF (right column) groups across the first several hours. All rats were presented both the chow and HE diets across these test sessions and comparisons between responses following 3.0 μg/kg CCK and the previous saline-injected session are shown in each graph. No significant differences between saline and CCK conditions were observed.

**Fig. 5.**
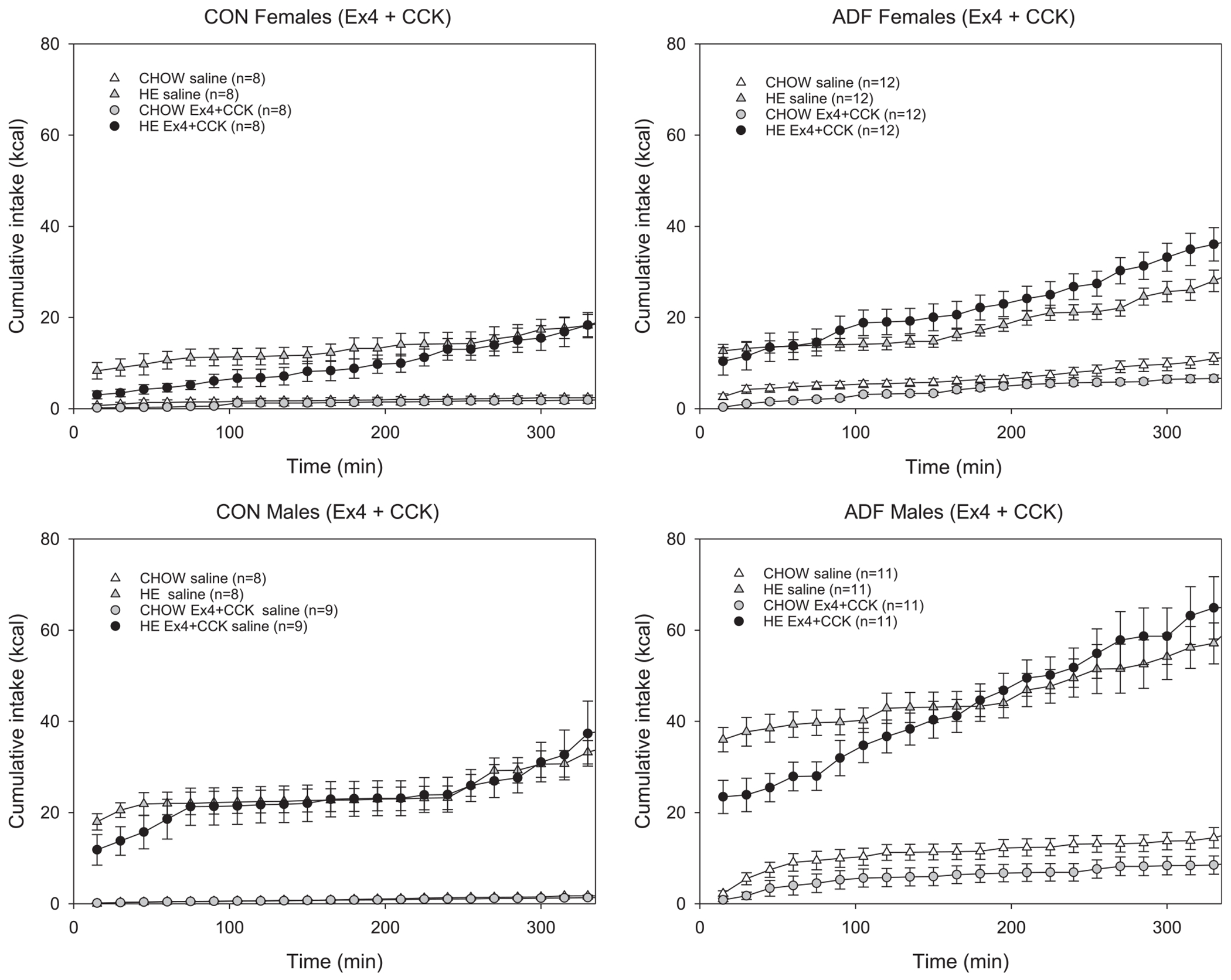
Cumulative caloric intake ± SE for chow and HE for female (top row) and male (bottom row) control (left column) and ADF (right column) groups across the first several hours. All rats were presented both the chow and HE diets across these test sessions and comparisons between responses following administration of 3.0 μg/kg Ex-4 and 3.0 μg/kg CCK.

To compare the effect of Ex-4, CCK and the combination of the two, cumulative intake on peptide-administration days was compared with the previous respective saline-injected test session. Based on the time course for the effects of Ex-4 and CCK on intake [21,45,55,79], cumulative intake across the first 330 min of the test session is shown (Figs. 3–5). To also account for changes earlier on the session, responses across the first 90 min and 330 min of an Ex-4, CCK or combination session were compared to those from the previous saline-injected condition. Compared to saline, 3 μg/kg Ex-4 decreased HE intake for male CON rats, but not females (Fig. 3). For female and male ADF groups, there were no main effects of 3 μg/kg Ex-4 on HE or chow intake compared to saline conditions. Within-subjects ANOVAs (condition × time) revealed a main effect of condition for male CON rats HE intake, but not at the earlier time point (Table 2), nor for other comparisons (Tables 2 and 3). An interaction effect for male ADF chow intake was revealed but post-hoc comparisons revealed no condition differences across any of the time points. For female and male rats, irrespective of experimental group, 3 μg/kg CCK did not affect intake in this paradigm (Fig. 4). Comparing intake between CCK and saline conditions revealed no main effect of condition for chow nor HE diet for any of the groups. Interaction effects for male CON chow intake (Table 2) and female CON chow intake (Table 3) were observed but no significant differences across any of the time points were revealed. For ADF rats, the combination of Ex-4 and CCK primarily resulted in lower cumulative intake of chow for the first hours (Fig. 5). Within-subject ANOVAs (condition × time) comparing chow intake across the first 90 and 330 min revealed main effects of Ex4 + CCK injection for female ADF and male ADF groups (Tables 2 and 3). For male ADF the combination also decreased HE intake but only earlier in the session (Fig. 5, Table 2). For female ADF rats, the combination resulted in reduced chow intake that was evident from the first 90 min followed by higher HE intake compared to the previous saline condition (Fig. 5, Table 3). In contrast, the administration of Ex-4 and CCK decreased HE diet intake for female controls but had no effect on intake for male control rats (Fig. 5, left panels).

### 4.3. Water intake

Water intake values on peptide-administration days were compared with that of each previous respective saline-injected test session. Paired t-tests revealed that female CON rats drank more water during the session following Ex-4 and CCK administration (24.8 ± 1.7 g) than during the preceding saline-injection test session (20.5 ± 1.4 g). Male CON rats tended to drink more water during the Ex-4 + CCK test session (26.5 ± 1.8 g) than the previous test session (24.6 ± 1.7 g) but this did not reach statistical significance. Water intake did not significantly differ between the other conditions compared (Table 4).

### 4.4. Estrous cycles

Comparing total caloric intake following administration of Ex-4, a one-way ANOVA revealed no significant group difference (F(1,19) = 0.730, p = 0.403). The similar group values remained when estrus was added as a dichotomous covariate (estrus vs. non-estrus; F(1,19) = 0.240, p = 0.630). Following administration of CCK, a one-way ANOVA comparing total caloric intake revealed no significant group difference (F(1,19) = 0.091, p = 0.766). Similar group values remained when estrus was added as a covariate (F(1,19) = 0.775, p = 0.390). Similarly, comparing total caloric intake following administration of Ex-4 and CCK, a one-way ANOVA revealed no significant group difference (F (1,18) = 0.206, p = 0.656). The similar group values remained when estrus was added as a covariate (F(1,18) = 0.394, p = 0.538).

## 5. Discussion

Compared to saline-control sessions, administration of the combination of Ex-4 and CCK, but not Ex-4 or CCK alone, decreased chow and HE intake early in the session for male ADF rats but primarily decreased chow intake for female ADF rats followed by an effect on HE diet intake. For the control groups, the combination resulted in a decrease in HE intake for female controls and had no effect on intake for males. Thus, when two diets are presented following 24-h food restriction, Ex-4 or CCK administration alone does not affect food intake in rats on an ADF schedule, but the combination produces decreases in feeding that are more than the sum of their individual effects. The changes in HE diet preference is primarily driven by increased chow intake in female and male ADF rats. Male ADF rats also decrease HE intake compared to same-sex controls. Given that the administration of Ex-4 + CCK combination decrease chow intake in both female and male ADF rats but have differential effects on HE intake, these findings support a role for the combination of GLP-1 and CCK signaling in the changes in diet preference induced by an alternate day fasting paradigm that differentially affects female and male rats.

Although the actions of various gut peptides in the regulation of feeding have been individually studied, feeding behavior does not alter the secretion of only a single gut peptide. A number of studies have examined the potential for synergistic effects among gut peptides. Combination injections of individually subthreshold doses produce decreases in feeding that are more than their individual effects [34,44]. Here, GLP-1 and CCK signaling appear to be differentially involved in ADF-induced diet preference in female and male rats. Published findings provide evidence that endogenous CCK interacts with GLP-1 signaling to promote satiation and that CCK receptor-expressing gastrointestinal vagal afferents play a primary role in these mechanisms [74]. A number of reports have identified CCK-expressing neurons in the caudal brainstem [10,14,60]. It has been demonstrated that these neurons are responsive to GLP-1 receptor agonists and are necessary for the food intake suppression and weight loss effects of GLP-1 receptor agonists [11]. In DIO rats, co-agonism of CCK and GLP-1 receptors induced greater weight loss and effects on food intake than the individual effects combined [73]. In mice, the combination of CCK and GLP-1 receptor agonism lowered glucose plasma levels and improved measures of glucose tolerance more so than treatment of CCK or GLP-1 receptor agonists alone [37]. Furthermore, a novel GLP-1 receptor and CCK-1 receptor co-agonist produced comparable or superior effects than semaglutide, a GLP-1 receptor agonist or NN9056, a CCK-1R agonist alone, in a number of metabolic measures including food intake, body weight and lipid regulation [78]. In contrast, it has been previously reported that twice daily injection of CCK receptor agonist alone or with GLP-1-receptor agonist decreased food intake, body weight gained and improved insulin sensitivity in high fat-fed mice but there were no differences between CCK treatment alone or in combination [37]. We previously published findings that support the role of GLP-1 alone, but not CCK alone or in combination with GLP-1, in taste-guided appetitive and consummatory behaviors to compounds described by humans as sweet and fatty [70]. Thus, the current findings indicate that the combination of GLP-1 and CCK signaling are involved in the ADF-induced changes in diet preference but more broadly, GLP-1 and CCK signaling play differential roles in various components of ingestive behavior.

At doses that have been shown to reduce chow intake, Ex-4 and CCK alone did not have robust inhibitory effects on chow or HE diet intake in ADF rats. Administration of 3 μg/kg Ex-4 reduced HE intake in male, but not female control rats. All groups in the current experiment were presented both chow and HE diet during test sessions in contrast to single diets presented in the majority of previous reports investigating food intake suppression with GLP-1 or CCK receptor agonism. The current findings indicate these doses are not as effective at reducing intake when chow and HE diet are simultaneously presented whether following food restriction or ad libitum access to chow. The dampened effect appears to be driven by increased intake with simultaneous diet presentation which is consistent with reports of increased overall intake when an array of food stimuli are presented than when diets are presented one at a time [72] or with the same components as a single diet [41]. Furthermore, suppression effects of peptides including GLP-1 and CCK appear to be less effective with increased exposure to a high energy diet. While GLP-1 receptor agonists similarly reduce intake of standard chow or high fat diet in rodents maintained on high fat diet for 19 days [31], rats maintained on high fat diet for 4–6 weeks fail to respond to GLP-1 whether tested on a low- or high-fat diet [77]. Similarly, in rats maintained on a high fat diet for several weeks [12,18,63,68] and in mice on an intermittent access schedule to a sucrose solution for several weeks [27], CCK administration is less effective in reducing food intake compared to controls. All groups in the current experiment had been exposed to the high energy diet for three 23-h sessions previously, thus it is possible that the lack of suppression effects of Ex-4 and CCK alone here is attributed by this diet exposure history and/or the presentation of two diet types in a test session. These possibilities are not mutually exclusive and raise the importance of further exploring the role of gut peptides with diet history and presentation variety as experimental variables, which provide more ecological validity.

The data indicate that there are sex differences in the underlying physiological mechanisms underlying changes in ADF-induced diet preference. Consistent with our previous findings [23], both female and male ADF groups ate more chow than their respective control groups, but male ADF rats also decreased HE diet intake. Here, we extend these findings to show that the combination of Ex-4 and CCK resulted in a decrease in HE intake for female controls yet had no significant effect on intake for the male control group. Co-agonism of CCK and GLP-1 receptors has been previously shown to reduce chow intake in male rats [73] and mice [78] yet in the current paradigm in which chow intake decreases with the addition of HE diet, the combination decreased HE intake in females but not males. For ADF groups, the combination decreased chow and HE intake early in the session for male ADF rats but primarily decreased chow intake for female ADF rats indicating sex differences in the gut peptide signaling related to this phenomenon. We previously published data in which this combination administered at the same dose and timing decreased chow intake in food-restricted male rats [70]. In the current experiment, the combination also affects HE diet intake although differently for female and male rats. Together these findings guide further investigation to more directly compare potential sex differences in the mechanisms that drive oral and postoral cues and mechanisms of reward and satiety. Here, when condition of estrus versus non-estrus was added as a covariate from measures of daily vaginal smears, no changes in group comparisons of overall caloric intake were observed indicating estrous cycle had no effect on overall intake. In contrast, it has been shown that changes in chow meal size as a function of estrous cycle is related to CCK signaling [20] and in ovariectomized rats, estradiol treatment increases the food suppression effects of CCK [26,47]. Given that in the current findings, CCK alone is insufficient to decrease intake in response to simultaneous presentation of chow and HE diet, it is possible that changes as a function of ovarian cycle do not explain the sex differences observed. However, without further experiments designed to directly address this, the possibility that changes across the estrous cycle affect other aspects of intake, particularly shorter-term cues that control meal parameters remains open.

Sex differences observed in the cumulative intake over time, point to the timing of gut peptide signaling. Following Ex-4 and CCK-induced suppression of intake early in the session, female ADF rats increased HE diet intake compared to saline-condition, during a time which peptide administration would not likely have a direct effect. These findings suggest female ADF rats compensated for the intake suppression earlier in the session. Similarly, it has been previously reported that food-restricted male mice that inhibited chow intake following CCK administration early in a test session, increased intake later in the session [74]. The doses and timing of administration of Ex-4 and CCK were chosen based on effects on intake of single diets (e.g. standard chow or calorically dense diets) [21,45,55,79]. It has been shown previously that Ex-4 for example decreases intake compared to vehicle-control at earlier (1 h) or later (2, 4, or 8 h) time points from injection, depending on the caloric density of the diet and previous diet exposure. Taking this into account, in the current experiment, Ex-4 and CCK were administered 3 h and 15 min respectively, before presentation of the two diets. We previously used this experimental design to compare effects on chow intake and taste-guided responses to sucrose and intralipid. However, these previous findings were obtained from experiments with male subjects and it is possible that there are sex differences in the timing of gut peptide signaling. The differential effects of Ex-4 and Ex-4 + CCK in the current experiment support this possibility. In contrast to single bolus intraperitoneal administration, compounds that affect appetite are often administered intravenously over 60–120 min before meal presentation in clinical settings [4,29] which may better account for individual variability. The current findings suggest that GLP-1 and CCK signaling play differential roles in ADF-induced changes in intake in female and male rats and highlight the importance of considering conditions that may affect individual variability in gut peptide signaling.

A growing body of evidence indicates that the effectiveness of gut peptides including CCK and GLP-1 to induce food intake suppression depends on energy homeostatic status. In the current experiment, the combination of Ex-4 and CCK differentially affected intake in CON and ADF groups that were tested in non-food-deprived and food-deprived states respectively. Previous reports in which food deprivation schedules blunt the food suppression effects of CCK in rats [50] and baboons [67] appears to at least be partially mediated by noradrenergic A2 neurons centrally [48]. Similarly, at doses that decrease food intake in a non-food-restricted state, GLP-1 does not reduce intake in rats [61] or mice [38,74] following a fasting period. Thus, it appears that CCK and GLP-1 inhibit food intake in postprandial, but not as effectively in food-deprived conditions. Food deprivation status also affects some aspects of the hedonic drives of ingestive behavior. When tested in a chow-fed condition, rats classically conditioned to associate distinct flavors earlier or later in a conditioning session, prefer the flavor associated with later than that associated with earlier in the session. Yet when tested in a food-deprived condition this preference was not observed [64]. Using an operant conditioning procedure, rats with experience of repeated cycles of restriction and refeeding to chow, display comparable lever presses for valued and devalued foods in contrast to the control group that respond more to the lever associated with the valued option [57]. Taken together, the current findings suggest the interaction of CCK and GLP-1 signaling differentially function depending on energy homeostatic condition.

Consistent to our previous report [23], change in diet preference in both female and male ADF rats was driven by increased chow compared to controls. Here, cumulative intake data reveal short- and long-term learning effects in ADF rats. Upon the first presentation of HE diet with the familiar chow, for ADF rats, caloric intake from both diets was comparable for the first several hours followed by hyperphagia of the HE diet suggesting that following 24-h food restriction, orosensory (e.g. high fat/high sugar oral cues) and postoral (e.g. calories) characteristics of the diets is learned during the first several hours of diet sampling. This phenomenon of diet sampling of familiar chow and novel HE is not observed in CON rats that when presented the novel HE diet in a nonfood restricted state showed almost exclusive preference for the HE diet. From the second test exposure onwards, ADF rats primarily ate the HE diet for the first ~ 10 h at a faster rate than the CON groups indicating changes in behavior as a consequence of the first test exposure. This phenomenon is consistent with previous findings in the literature demonstrating changes in flavor preference as a function of food-deprivation [5,43]. Furthermore, although the changes in pattern of intake from novel to familiar HE diet are observed in both female and male groups, some sex differences in rate of intake may point to sex differences in the underlying mechanisms that influence ingestive behavior such as oral and postoral cues. In the current experiment, in light of the evidence for learning across test sessions, the effects of Ex4, CCK and the combination were compared with responses during each previous saline-administered session. To assess the effect of Ex4 and CCK on responses to novel diet, it would be important to conduct experiments to compare responses in saline-injected and agonist-injected groups. Association of the orosensory and/or postoral features of the diet likely explain the changes in cumulative intake curves across test sessions.

All groups show a preference for the HE diet over the less calorically dense standard chow, yet several hours into the test sessions, ADF eat both HE and chow diets which may indicate aspects of sensory specific satiety in which intake of the current food declines, but intake increases when presented a different food. Evidence in the literature indicate that in male rats, repeated cycles of food restriction and refeeding, but not continuous food restriction disrupts sensory specific satiety [1,57]. It has been proposed that intermittent fasting is a type of stressor and that the disruption of sensory specific satiety following repeated intermittent fasting periods is akin to the cross-sensitization to psychostimulants following repeated exposures to stressors. Indeed, it has been reported that repeated exposure to restraint [3] or tail-pinch [32] stressors in male rats increases food intake across test sessions. This phenomenon may at least partially explain the increased chow observed in female and male ADF rats.

The combination of GLP-1 and CCK signaling appear to be involved in the underlying physiological mechanisms that drive changes in diet intake induced by an alternate day fasting paradigm. The current findings raise the importance of considering diet exposure history and further exploring the similarities and sex differences that food-restriction paradigms have on ingestive behavior.

## Figures and Tables

**Table 1:** Injection schedule.

Test day	Injection (ml/kg)	Before chow and HE diet presentation
1	saline	15 min
2	saline	15 min
3	saline	3 h
4	3 μg/kg Ex-4	3 h
5	saline	15 min
6	3 μg/kg CCK	15 min
7	saline	3 h
	saline	15 min
8	3 μg/kg Ex-4	3 h
	3 μg/kg CCK	15 min

**Table 2:** Comparisons of chow and HE intake for male CON and ADF groups across first 330 and 90 min on Ex-4, CCK and Ex-4 + CCK administration test days with food intake during previous respective saline-injection days.

	Time (min)	Ex-4	CCK	Ex-4 + CCK
CON m chow	330	Condition (F(1,11) = 0.038, p = 0.849, Time (F(21,231) = 6.050, p < 0.001, C × T (F(21,231) = 0.962, p = 0.512	Condition (F(1,10) = 2.428, p = 0.150, Time (F(21,210) = 37.785, p < 0.001, C × T (F(21,210) = 2.376, p = 0.001	Condition (F(1,7) = 0.403, p = 0.546, Time (F(21,147) = 45.407, p < 0.001, C × T (F(21,147) = 1.121, p = 0.333
	90	Condition (F(1,11) = 0.023, p = 0.883, Time (F(5,55) = 8.890, p = 0.003, C × T (F(5,55) = 0.259, p = 0.934	Condition (F(1,10) = 0.479, p = 0.505, Time (F(5,50) = 5.522, p < 0.001C × T (F(5,50) = 2.249, p = 0.064	Condition (F(1,7) = 0.016, p = 0.903, Time (F(5,35) = 35.572, p < 0.001, C × T (F(5,535) = 0.655, p = 0.660
CON m HE	330	Condition (F(1,11) = 6.027, p = 0.032, Time (F(21,231) = 25.620, p < 0.001, C × T (F(21,231) = 1.187, p = 0.264	Condition (F(1,10) = 0.046, p = 0.834, Time (F(21,210) = 18.866, p < 0.001, C × T (F(21,210) = 0.299, p = 0.999	Condition (F(1,7) = 0.093, p = 0.769, Time (F(21,147) = 13.216, p < 0.001, C × T (F(21,147) = 1.244, p = 0.224
	90	Condition (F(1,11) = 1.169, p = 0.303, Time (F(5,55) = 3.045, p = 0.017, C × T (F(5,55) = 0.345, p = 0.883	Condition (F(1,10) = 0.626, p = 0.447, Time (F(5,50) = 7.686, p < 0.001C × T (F(5,50) = 0.057, p = 0.998	Condition (F(1,7) = 2.283, p = 0.175, Time (F(5,35) = 5.094, p < 0.001, C × T (F(5,535) = 1.143, p = 0.356
ADF m chow	330	Condition (F(1,11) = 0.116, p = 0.740, Time (F(21,231) = 11.557, p < 0.001, C × T (F(21,231) = 2.846, p < 0.001	Condition (F(1,11) = 1.156, p = 0.305, Time (F(21,231) = 12.476, p < 0.001, C × T (F(21,231) = 0.374, p = 0.995	Condition (F(1,10) = 9.401, p = 0.012, Time (F(21,210) = 12.476, p < 0.001, C × T (F(21,210) = 1.018, p = 0.443
	90	Condition (F(1,11) = 0.649, p = 0.437, Time (F(5,55) = 4.143, p = 0.003, C × T (F(5,55) = 0.227, p = 0.949	Condition (F(1,11) = 2.113, p = 0.174, Time (F(5,55) = 8.685, p < 0.001C × T (F(5,55) = 0.518, p = 0.762	Condition (F(1,10) = 11.509,p = 0.007, Time (F(5,50) = 9.942, p < 0.001, C × T (F(5,50) = 2.084, p = 0.083
ADF m HE	330	Condition (F(1,11) = 0.013, p = 0.913, Time (F(21,231) = 28.966, p < 0.001, C × T (F(21,231) = 1.139, p = 0.309	Condition (F(1,11) = 0.720, p = 0.414, Time (F(21,231) = 27.718, p < 0.001, C × T (F(21,231) = 1.151, p = 0.297	Condition (F(1,10) = 0.549, p = 0.476, Time (F(21,210) = 20.217, p < 0.001, C × T (F(21,210) = 3.905, p < 0.001
	90	Condition (F(1,11) = 1.164, p = 0.304, Time (F(5,55) = 8.176, p < 0.001, C × T (F(5,55) = 1.900, p = 0.109	Condition (F(1,11) = 1.661, p = 0.224, Time (F(5,55) = 8.305, p < 0.001C × T (F(5,55) = 1.283, p = 0.284	Condition (F(1,10) = 11.874,p = 0.006, Time (F(5,50) = 9.710, p < 0.001, C × T (F(5,50) = 1.697, p = 0.153

**Table 3:** Comparisons of chow and HE intake for female CON and ADF groups across first 330 and 90 min on Ex-4, CCK and Ex-4 + CCK administration test days with food intake during previous respective saline-injection days.

	Time (min)	Ex-4	CCK	Ex-4 + CCK
CON f chow	330	Condition (F(1,10) = 1.806, p = 0.209, Time (F(21,210) = 73.781, p < 0.001, C × T (F(21,210) = 1.590, p = 0.054	Condition (F(1,9) = 1.562, p = 0.243, Time (F(21,189) = 4.541, p < 0.001, C × T (F(21,189) = 1.661, p = 0.040	Condition (F(1,7) = 0.934, p = 0.366,Time (F(21,147) = 9.599, p < 0.001, C × T (F(21,147) = 0.432, p = 0.986
	90	Condition (F(1,10) = 0.795, p = 0.394,Time (F(5,50) = 8.824, p < 0.001, C × T (F(5,50) = 0.336, p = 0.889	Condition (F(1,9) = 1.596, p = 0.238,Time (F(5,45) = 3.271, p = 0.013, C × T (F(5,45) = 2.413, p = 0.051	Condition (F(1,7) = 2.297, p = 0.173,Time (F(5,35) = 6.016, p < 0.001, C × T (F(5,35) = 1.257, p = 0.304
CON f HE	330	Condition (F(1,10) = 0.438, p = 0.523,Time (F(21,210) = 34.683, p < 0.001, C × T(F(21,210) = 0.409, p = 0.991	Condition (F(1,9) = 0.270, p = 0.616, Time (F(21,189) = 17.725, p < 0.001, C × T (F(21,189) = 0.962, p = 0.513	Condition (F(1,7) = 3.299, p = 0.112,Time (F(21,147) = 24.067, p < 0.001, C × T (F(21,147) = 2.369, p = 0.001
	90	Condition (F(1,10) = 0.316, p = 0.586,Time (F(5,50) = 5.470, p < 0.001, C × T (F(5,50) = 0.790, p = 0.562	Condition (F(1,9) = 0.156, p = 0.702,Time (F(5,45) = 13.331, p < 0.001, C × T (F(5,45) = 0.770, p = 0.577	Condition (F(1,7) = 12.163, p = 0.010,Time (F(5,35) = 6.544, p < 0.001, C × T (F(5,35) = 0.131, p = 0.984
ADF f chow	330	Condition (F(1,10) = 0.268, p = 0.616,Time (F(21,210) = 21.474p < 0.001, C × T (F(21,210) = 0.505, p = 0.966	Condition (F(1,11) = 1.736, p = 0.214, Time (F(21,231) = 51.143, p < 0.001, C × T (F(21,231) = 0.679, p = 0.852	Condition (F(1,11) = 7.746, p = 0.018, Time (F(21,231) = 47.208, p < 0.001, C × T (F(21,231) = 1.333, p = 0.155
	90	Condition (F(1,10) = 0.749, p = 0.407,Time (F(5,50) = 10.848, p < 0.001, C × T (F(5,50) = 0.408, p = 0.841	Condition (F(1,11) = 1.145, p = 0.308,Time (F(5,55) = 23.717, p < 0.001, C × T (F(5,55) = 0.816, p = 0.543	Condition (F(1,11) = 3.299, p = 0.002,Time (F(5,55) = 19.121, p < 0.001, C × T (F(5,55) = 1.288, p = 0.282
ADF f HE	330	Condition (F(1,10) = 0.526, p = 0.485,Time (F(21,210) = 31.683, p < 0.001, C × T (F(21,210) = 1.490, p = 0.083	Condition (F(1,11) = 0.226, p = 0.644, Time (F(21,231) = 38.792, p < 0.001, C × T (F(21,231) = 1.138, p = 0.310	Condition (F(1,11) = 5.608, p = 0.037, Time (F(21,231) = 31.241, p < 0.001, C × T (F(21,231) = 3.403, p < 0.001
	90	Condition (F(1,10) = 1.387, p = 0.266,Time (F(5,50) = 11.127, p < 0.001, C × T (F(5,50) = 4.770, p = 0.832	Condition (F(1,11) = 1.479, p = 0.249,Time (F(5,55) = 15.244, p < 0.001, C × T (F(5,55) = 0.349, p = 0.881	Condition (F(1,11) = 0.001, p = 0.981,Time (F(5,55) = 10.285, p < 0.001, C × T (F(5,55) = 3.969, p = 0.004

**Table 4:** Comparisons of water intake for each group on Ex-4, CCK and Ex-4 + CCK administration test days with water intake during previous respective saline-injection days.

	Ex-4	CCK	Ex-4 + CCK
ADF f	t(11) = −0.168, p = 0.870	t(11) = −1.229, p = 0.245	t(11) = 1.074, p = 0.306
CON f	t(11) = −1.341, p = 0.207	t(11) = −1.011, p = 0.334	t(11) = −0.463, p = 0.001
ADF m	t(11) = −1.150p = 0.275	t(11) = −0.513, p = 0.618	t(11) = 0.464, p = 0.652
CON m	t(11) = −1.648, p = 0.128	t(11) = −0.117, p = 0.909	t(11) = −1.962, p = 0.076

## Data Availability

Data will be made available on request.
